# Public awareness of the EMS system in Western Saudi Arabia: identifying the weakest link

**DOI:** 10.1186/s12245-015-0070-7

**Published:** 2015-09-07

**Authors:** EMSAT: The Emergency Medical Service Assessment Team group, A F Hamam, M H Bagis, K AlJohani, A H Tashkandi

**Affiliations:** Emergency Department, King AbdulAziz Medical City, MNGHA, Jeddah, Saudi Arabia; Emergency Department, King Fahad Medical City, MNGHA, Riyadh, Saudi Arabia; Emergency Department, Prince Mohammad Bin AbdulAziz Medical Center, MNGHA, Madinah, Saudi Arabia; McMaster University, Hamilton, ON Canada

## Abstract

**Background:**

The City of Jeddah is the major and largest city in the Western Region of the Kingdom of Saudi Arabia (KSA). Covering a total area of 748 km2. The Saudi Red Crescent Organization (SRC) makes up the major bulk of the Emergency Medical Service (EMS) system in the Kingdom. We have set out to investigate the level of public awareness of the EMS system in place in Western KSA.

**Method:**

This study was an observational cross-sectional study that was done by interviewing the general public in public venues. The survey consisted of a two part questionnaire. The first part was completed for all subjects. The second part was completed only for those subjects that had previous experience with the SRC service.

**Result:**

A total of 1534 subjects were interviewed by 5 data collectors. 33% of people did not know the emergency dispatcher number to call in case of a medical emergency. The majority estimated the ETA of an ambulance response to their home to be about 30 minutes or more. 94 % said that MEDEVAC is needed. 17.7 % of people still find it unacceptable for male paramedics to respond to a female emergency unescorted by a male family member.

**Conclusion:**

It is clear that the general public is aware of the deficit in EMS coverage that is present. To improve the public awareness of the EMS system, municipal, legislative, public guidance, as well as religious support, are needed to be utilized to improve the community’s satisfaction and quality of care.

**Electronic supplementary material:**

The online version of this article (doi:10.1186/s12245-015-0070-7) contains supplementary material, which is available to authorized users.

## Background

The City of Jeddah is the major and largest city in the Western Region of the Kingdom of Saudi Arabia (KSA). Covering a total area of 748 km, it is considered the major harbor on the Red Sea and is the main entry point to the annual pilgrimage to Makkah each year (*Hajj*). This large metropolitan, is serviced by the Saudi Red Crescent (SRC) organization which makes up the major bulk of the emergency medical service (EMS) system in the Kingdom.

The SRC has a fleet of 13 emergency units in the form of type II ambulances (van type), manned by EMT-B personnel (usually 2), and 6 advanced life support (ALS) units, manned by senior paramedics (usually 2), servicing a total of 3.4 million people as of October 2010. This EMS force is expandable to 27 units as the need arises. The SRC operates from a total of 13 base stations scattered throughout the city. During *Hajj* season, and in times of major disasters, the Saudi Civil Defense Force (SCDF) also aids in the victim rescue; however, the day-to-day EMS operations are carried out exclusively by the SRC.

To date, there has not been any assessment to ascertain the public awareness and expectations of the SRC. We do recognize that public awareness of how the EMS system works in KSA, and the ways to access it, is probably the most important link in the chain of survival of medical emergency and trauma victims. To that end, we have set out to investigate the level of public awareness of the EMS system in place in KSA, what the public thinks of the service that is being provided, and what their expectations are of this system. This information is crucial if we are to improve the SRC’s public image, identify the myths and misconceptions people have about the EMS system, and improve the overall satisfaction of the community.

## Methods

This study was an observational cross-sectional study that was done through an interviewed two-part questionnaire to the participating subjects asking them about their awareness, knowledge, and opinion of the current practices of the SRC. The questionnaire interview was conducted by PGY-2 emergency medicine residents, who have had a 4-h training session on how to ask the questions and conduct the interview. This was done in two parts. The first part was made up of 15 questions that collected the demographical data of the participant, general knowledge of the participant about multiple issues that pertain to the SRC, and whether or not they had previously made use of the SRC service before. Participants that have had previous experience with the SRC before went on to the second part of the questionnaire that asked them about that experience.

The first part of the questionnaire (Additional file 1) used partially validated questions from a previous questionnaire used in a previous EMS study done in 2002 [1]. Other questions, which addressed more local and cultural issues, were added in, based on previous reports filed by the practicing paramedics to their command, as being problematic or issues of concern over the time period of 3 years from 1 Jan. 2007 to 31 Dec. 2009.

Inclusion criterion for this study was being a member of the general public living within the city limits of Jeddah. Subjects excluded from participating in this survey were all individuals that were involved in the medical or paramedical field in any capacity (physicians, nurses, medical institutional clerks, paramedics, etc.), living outside the city limits, and being part of the Saudi Red Crescent organization or the Saudi Civil Defense Force in any shape or form. Interviews for this study were done in many public places such as shopping malls, banks, cafes, on the street, in the OPD waiting rooms of four major governmental hospitals, and major chain supermarkets. The sample size for this survey was calculated for a confidence level of 99 % and a tolerated error of 3.5 %, and in the general population of the city of Jeddah being 3,400,000 according to the 2009/2010 national municipal survey, our sample size was calculated to be 1,350.

Once all the data was collected, the information was input into a Microsoft Access database and analyzed. We thought that the main factor that would probably influence the opinion of the public about the SRC practices was having previous or firsthand experience with them. So with that in mind, we also set off to do a subgroup analysis to find out if the results we obtained were coincidental or if having previous SRC encounters influenced the publics’ answers. Hence, all answers to the first part questionnaire were analyzed for a significant *p* value using the *χ*^2^ test.

## Results

Between 1 July 2010 and 31 Dec. 2010, a total of 1551 subjects were interviewed by 5 data collectors. Of the 1551 interviews done, 17 interviews were not completed due to the subject interviewed being in a hurry and/or refusing to continue the questionnaire. That gave us a response rate of 98.9 %. Of the 1534 participants, 355 were found to have had previous experience with the SRC.

**Fig. 1:**
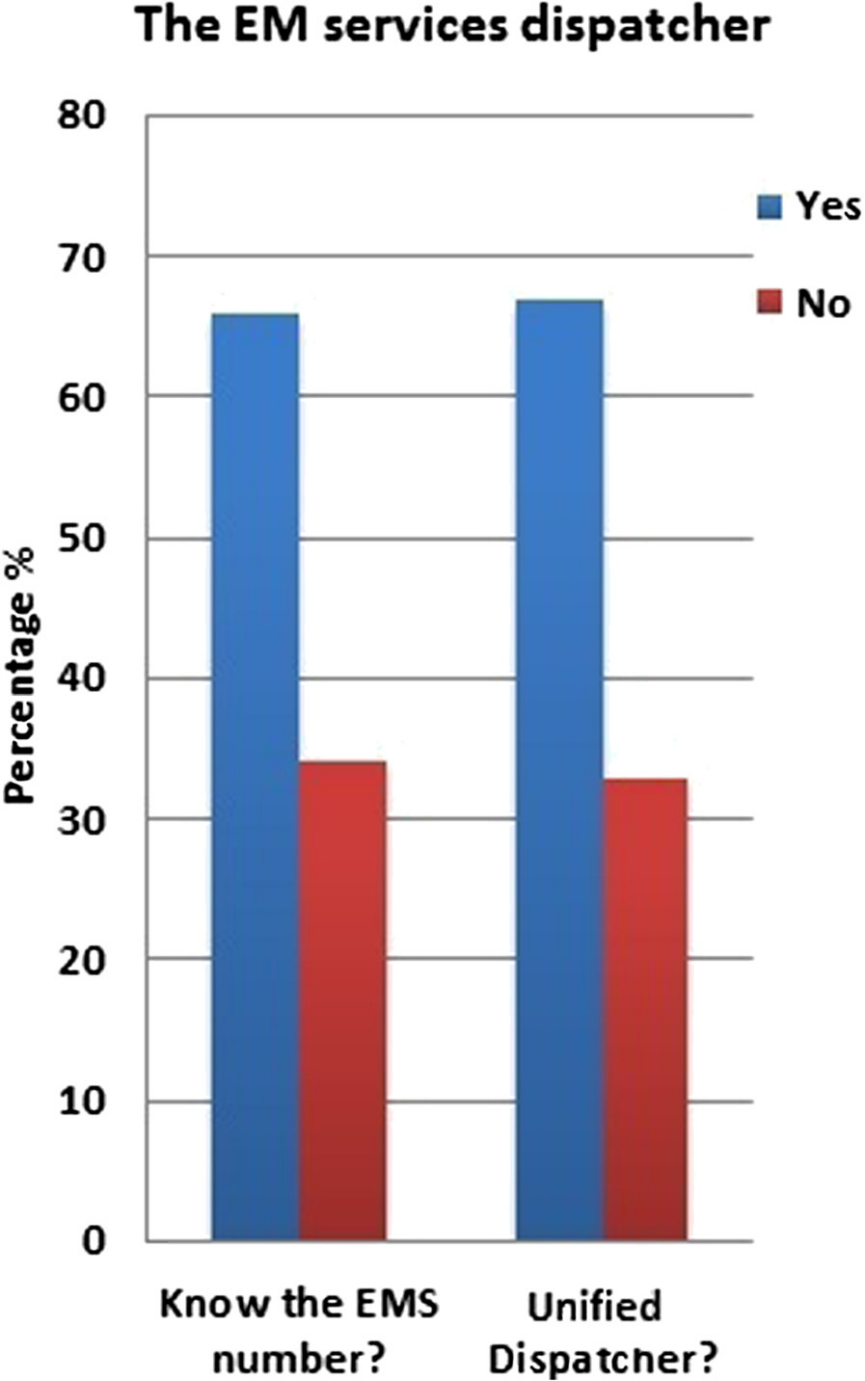
The EM service dispatcher

As is evident in Table 1, the majority of our sample was in the 15–30 years of age range, and 78.9 % was of Saudi nationality. Of the total sample, 23.1 % has had a previous experience with the SRC. The first and most important question of our survey was to know what percentage of the general public knew the emergency number to call (the dispatcher number) in case of a medical emergency. Our study showed that one out of three people only knew the emergency dispatcher number (Fig. 1). Interestingly, only 64.2 % (*n* = 328) of those that did not know the emergency dispatcher number said that they prefer the emergency services (EMS, police rescue, fire department, etc.) to be under a single dispatcher number (similar to 911 in the US).

**Table 1:** Demographics of public surveyed (*n* = 1534)

Sex	
Male	880 (57.3 %)
Female	654 (42.7 %)
Nationality	
Saudi	1211 (78.9 %)
Non-Saudi	323 (21.1 %)
Age	
15–30 years	816 (53.2 %)
30–45 years	487 (31.7 %)
45–60 years	181 (11.8 %)
60–75 years	30 (2.0 %)
> 75 years	20 (1.3 %)
Previous SRC encounter	
Yes	355 (23.1 %)
No	1179 (76.9 %)

**Fig. 2:**
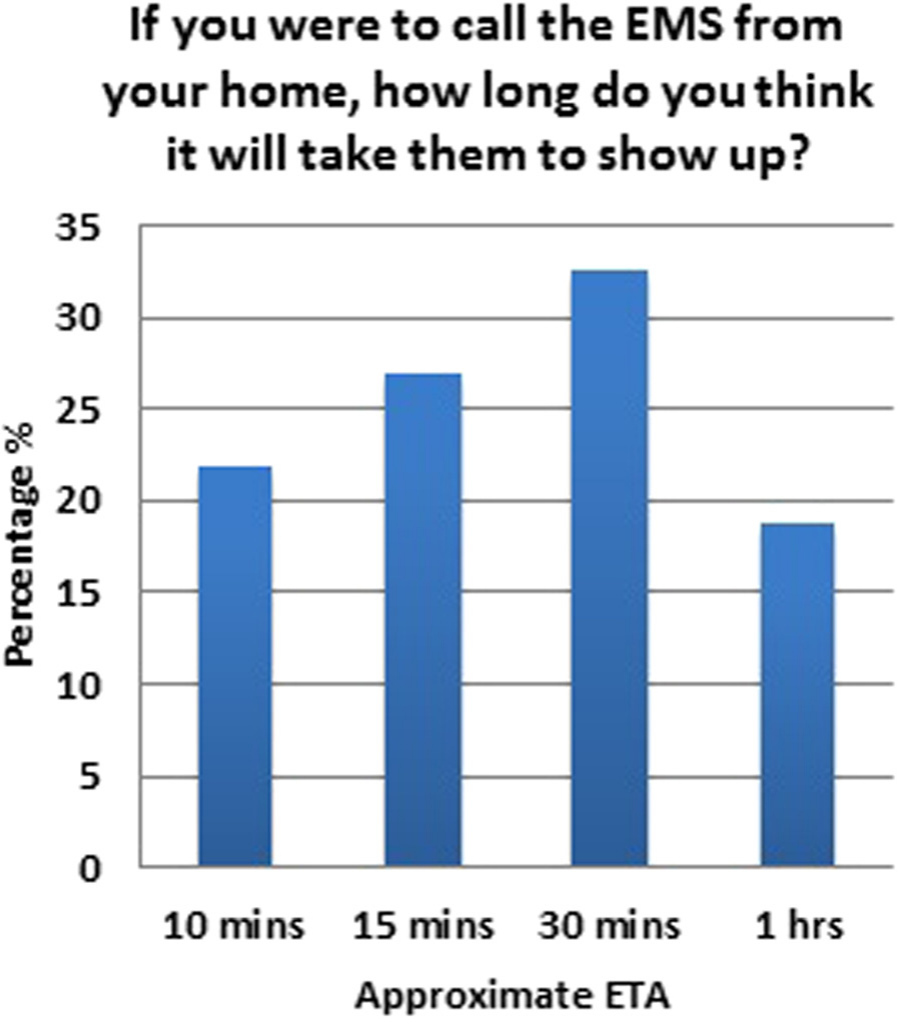
ETA for EMS if a person calls from home

Subjects enrolled in this study were asked to estimate that if they were to call an ambulance from their home, wherever that may be, how long would it be before the SRC ambulance showed up at their home. As is shown in Fig. 2, 32.4 % (*n* = 498) of people do not expect the ambulance to show up before 30 min, while another 18.8 % (*n* = 289) expect to wait 1 h before any help shows up. Of those that think that the SRC ETA is 1 h, 94.5 % (*n* = 273) think that there is not enough EMS coverage of the city and that MEDEVAC (the air ambulance) is needed to solve the problem. Overall, only 22 % (*n* = 347) of the total subjects thought that the SRC coverage of the city was adequate as it stands. Of the total population surveyed, only 8.4 % (*n* = 129) said that MEDEVAC was not needed.

**Fig. 3:**
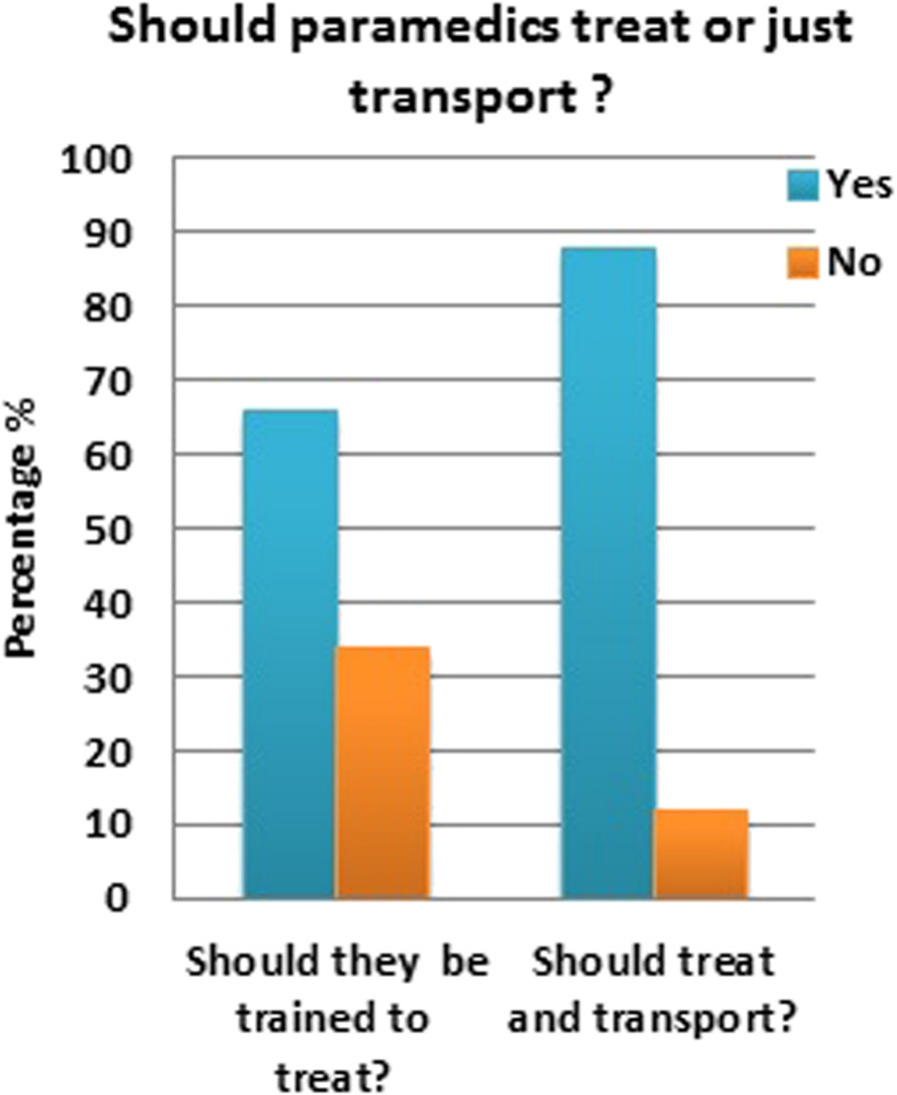
Expected role of paramedics

Another issue that was worthy of investigation was the trust the public had in the ability of the SRC personnel to treat patients at the scene. People were asked whether they thought that EMS personnel should be trained to treat patients on site or if their job was confined to merely facilitating the patient’s rapid transport to the nearest emergency department to receive medical care. As presented in Fig. 3, the majority of people did think that it was the duty of the paramedics to treat people on site, and that they should be trained, to offer life saving measures, not to just act as medical taxi drivers to get patients to the closest medical facility as soon as possible.

More towards the traditional customs in a patriarchal society, it was important for us to know what the general public thought of the situation where a male paramedic was responding to an emergency call at a home that contained only females, and no male escort (from the family) was present. In such a situation, did the public consider it acceptable for the male (stranger) to enter a house with no man present? Such questions might seem simple and obvious to western or medical minds. Despite there being a strict Islamic religious license that would allow the male paramedic to respond to a female medical emergency, of the people surveyed, up to 17.7 % (*n* = 272) still cling to cultural traditions, which have no foundation in religion, thinking that if there was no man in the house, paramedics should *not* enter to save a woman in a medical emergency.

With regards to the role that the EMS play in the medical system, participants in this survey were also asked who should they call in the event an elder person died at home (due to natural deterioration of health due to age or chronic conditions), 57 % (*n* = 874) said that they would call the SRC, while 26 % (*n* = 398) said that they would call the police to report the death. Only 17 % (*n* = 262) knew that they should call the municipal services to come and remove the body, as is the regulations in KSA.

Over the past years, we have noticed that on some occasions, the SRC has been called upon to transport patients that were not in medical emergency [2]. Examples of that were patients with mild abdominal discomfort, who are able to walk and could have got to the emergency room in their private car, or bedridden patients that have to get to the hospital to attend an outpatient appointment. Further to that point, we asked the participants in this survey if they thought that it was appropriate for the EMS personnel to refuse transport of patients if they were not in a medical emergency. Sixty-three percent (*n* = 967) of people surveyed agreed that engaging the EMS personnel with non-urgent transport was not only wrong but also irresponsible and somewhat immoral.

**Fig. 4:**
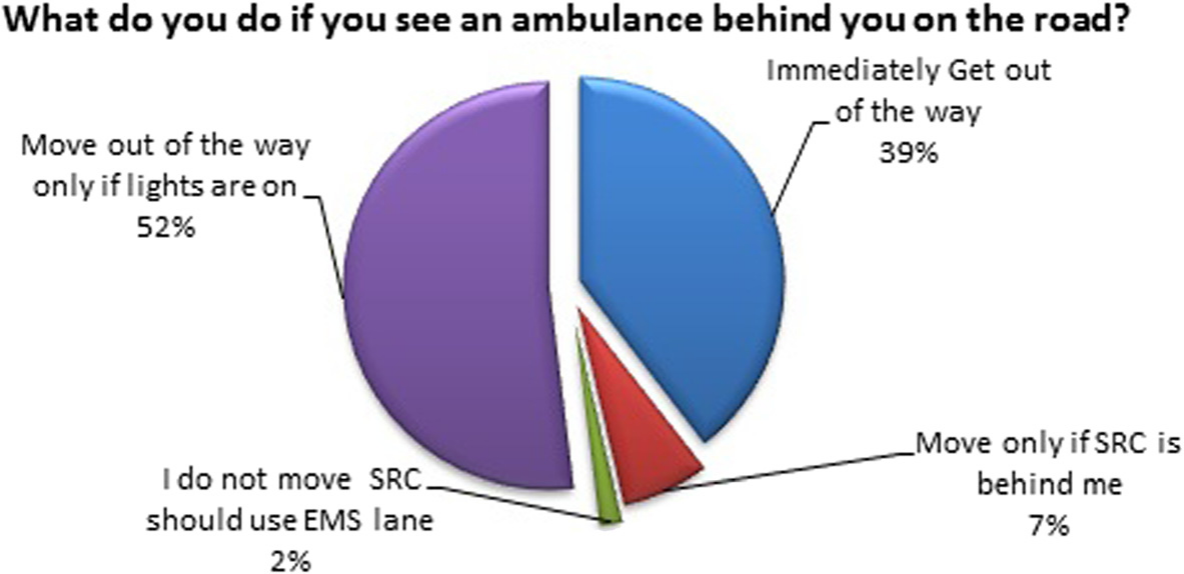
Response when an ambulance is on the road

Thirty-seven percent (*n* = 567) however, thought that once the paramedics arrive to assess a patient, they should not abandon him/her, regardless of whether he/she was in an emergency or not. In contrast, subjects were asked what they would do if they saw an ambulance in their rearview mirror gaining up on them on the road. Most people said that they would get out of the way immediately to allow the ambulance to pass, while others said that they would only move out of the way if the ambulance had its lights and sirens on, see Fig. 4.

**Fig. 5:**
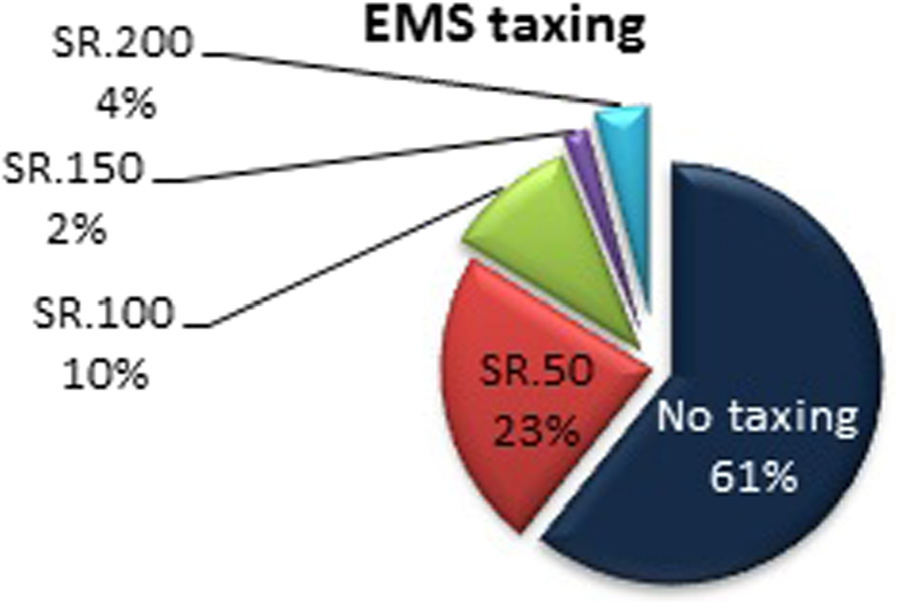
EMS taxing

**Fig. 6:**
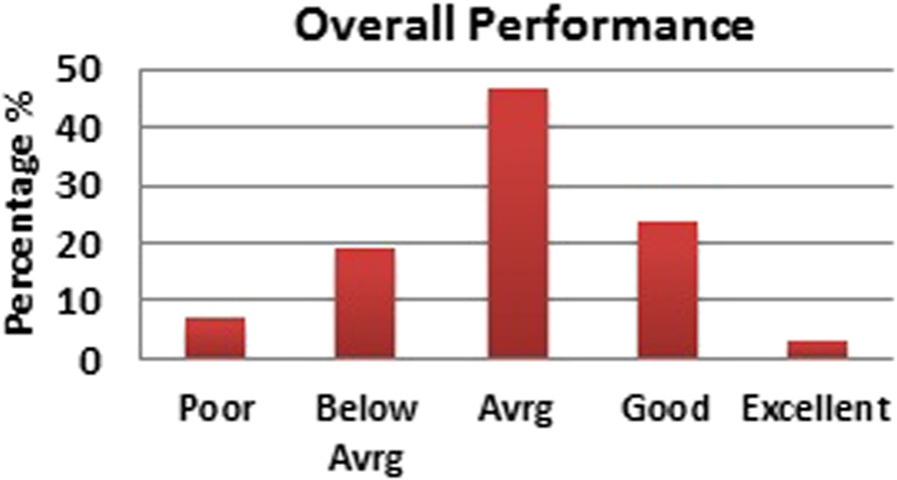
Overall performance of SRC

The EMS service in KSA is completely government funded. However, many people have voiced an opinion that for service to improve, some measure of taxing should be instituted in favor of the SRC. Similar to funding highway maintenance with tolls and taxes, we asked if some monetary sum should be collected annually (e.g., when a citizen renew his/her driver’s license) and how much they thought they would be willing to contribute. As Fig. 5 shows, the majority of people think that all EMS service in KSA should remain exclusively government funded, although one in three people thought that funding the SRC will improve their service. As far as the overall performance of the SRC, according to all subjects surveyed, those that had firsthand experience, and those that did not, 59.2 % (*n* = 907) of people say that they trust the SRC to handle medical emergencies. As shown in Fig. 6, the majority of people think that the performance of the SRC is average overall.

Subgroup analysis of those subjects with firsthand experience with the SRC, 23.1 % (*n* = 355), was performed, and a chi-squared test was conducted to ascertain whether answers to the information already mentioned above was dependent on whether or not the subject has had previous dealings with the SRC. As shown in Table 2, the *p* value was only significant for two questions: whether the subject knew the EMS dispatcher number, and whether or not there should be a unified emergency services number to call for all services (EMS, police rescue, fire department, etc.).

**Table 2:** Independence of answers with respect to the previous SRC encounter

	No previous SRC encounter		Previous SRC encounter		*p* value
	Yes	No	Yes	No	
Do you know the number to call in an emergency?	710 (46.3 %)	469 (30.6 %)	313 (20.4 %)	42 (2.7 %)	*<0.0001*
Should all emergency services be under one administration?	797 (52.0 %)	382 (25.0 %)	217 (14.1 %)	138 (8.9 %)	*0.02390*
Should paramedics treat patients in the field?	1035 (67.5 %)	144 (9.3 %)	321 (21.0 %)	34 (2.2 %)	0.17393
Do you think Jeddah has enough EMS coverage?	270 (17.6 %)	909 (59.2 %)	77 (5.0 %)	278 (18.2 %)	0.63267
Do you think we need the MEDEVAC?	1076 (70.1 %)	103 (6.7 %)	329 (21.4 %)	26 (1.8 %)	0.4006
Do you trust the EMS?	699 (45.6 %)	480 (31.3 %)	208 (13.6 %)	147 (9.7 %)	0.81510
Should paramedics enter a house with no male escort to respond to an emergency?	964 (62.8 %)	215 (14.0 %)	298 (19.5 %)	57 (3.7 %)	0.34590
Should paramedics be able to refuse transport of non-urgent cases?	741 (48.3 %)	438 (28.6 %)	226 (14.7 %)	129 (8.4 %)	0.7811
Should the SRC services be funded by the community?	463 (30.2 %)	716 (46.7 %)	139 (9.1 %)	216 (14.0 %)	0.9688

**Fig. 7:**
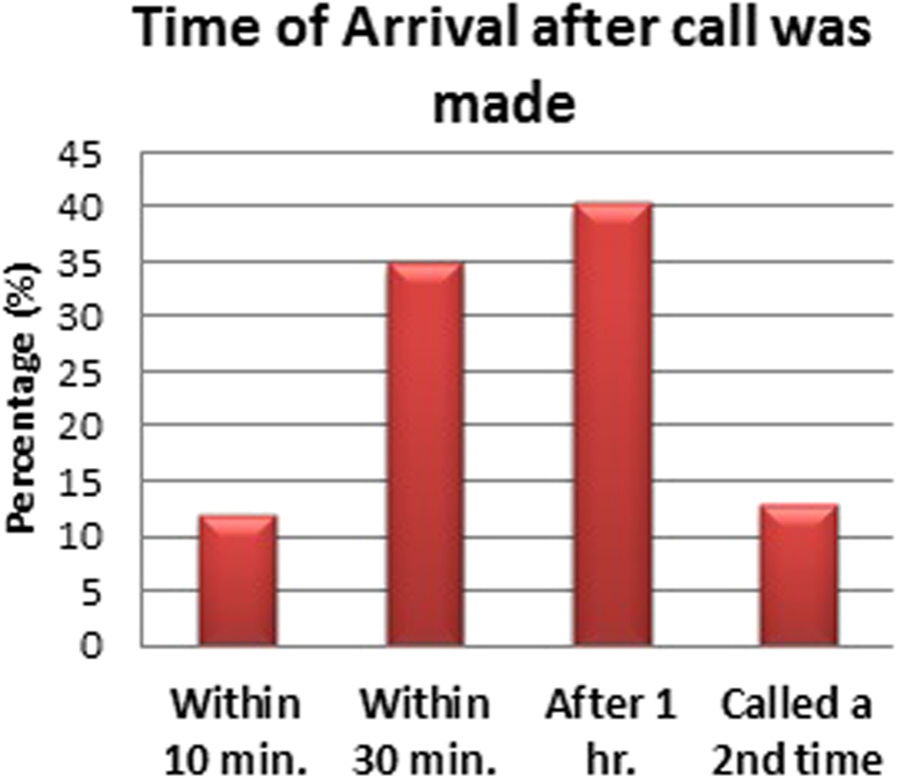
Time of arrival after call was made

Subjects that had previous experience with the SRC were asked six further questions, (Additional file 1), to ascertain their satisfaction with the EMS encounter and the service that was provided. As Fig. 7 illustrates, in 40.3 % (*n* = 143) of EMS responses, the SRC personnel arrived late (after 1 h), while in 13.0 % (*n* = 46), subjects reported that they had to call the SRC dispatcher a second time to ask about the ambulance that never arrived.

**Fig. 8:**
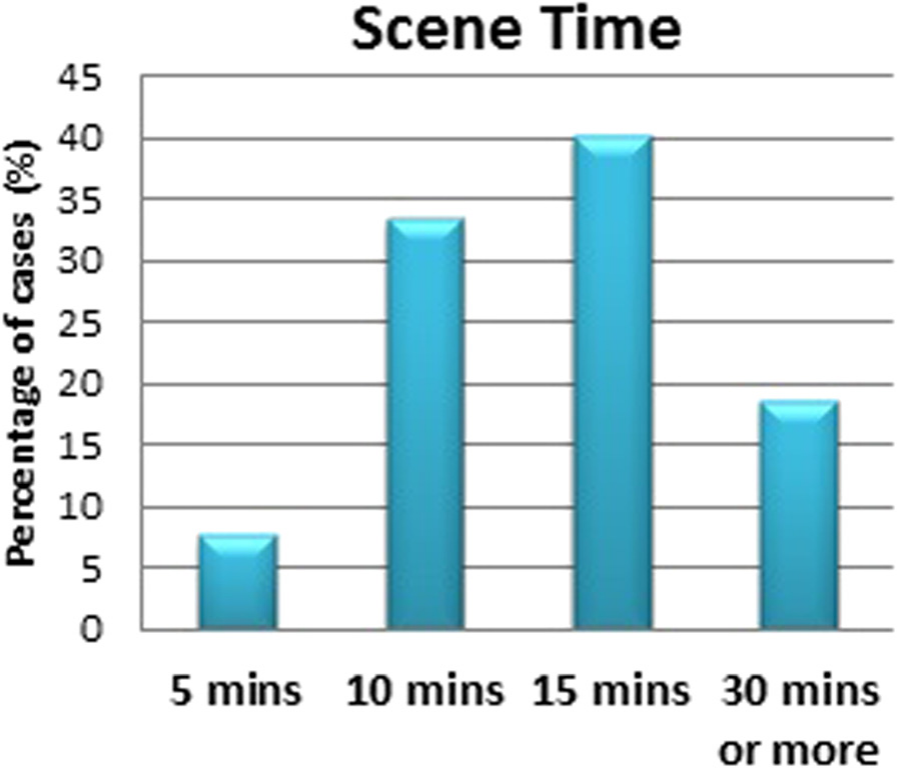
Scene time

On arrival, subjects reported that in 96.3 % (*n* = 342) of the time, SRC were in their official uniforms, and in 86.7 % (*n* = 308) of cases, they used gloves when they were handling the patients. In 52.1 % (*n* = 185) of cases, the paramedics attempted treatment in the field (treatment being anything, from putting on an oxygen mask to establishing and IV line and performing CPR). The most common scene time was 15 min as shown in Fig. 8. Finally, of those who actually called for the SRC services, 69.5 % (*n* = 247) reported that the paramedics did a good job overall.

## Conclusions and discussion

If public awareness is to be improved, the first step is to find out what the public knows and does not know about the EMS system in the Kingdom of Saudi Arabia. This survey represents an important step in uncovering what the public knows about the emergency medical services in this region of the country and what misconceptions they have. As mentioned above, only a third of the population is aware of the emergency dispatcher number. This of course has profound ramifications on the EMS system, since the majority of the general public are unable to access the system because they simply do not know what number to call [1, 3]. Hence, a campaign to advertise the number in hospital waiting rooms, television adverts, symposia, public boards, etc. is a first step in getting the general public to know the emergency number to call. A second step would be to unify the emergency services dispatcher number in order to reduce the information that any one individual should know. Thus, instead of knowing 999 for police and rescue, 998 for the fire department, 997 for EMS, 994 for traffic police, etc., a single number for all (similar to 911 in the US), after which, the call can be directed to the appropriate service. Such a feat will obviously take a lot of coordination to bring all these sectors together under one roof.

One major concern that seemed to stand out throughout this survey was the concern that the SRC takes a long time to arrive to the emergency scene. This has been shown to be consistent in both groups in this survey, those that did and did not have previous experience with the SRC. Most people have put the ETA for an ambulance to arrive around 30 min or so, and those who have actually used the SRC service before have found that they have arrived within 30 min in 50 % of cases, while they needed an hour in about 40 %, and in the remaining 10 %, they never showed up and had to be called again. Obviously, this is due to a number of factors such as (1) badly addressed districts, (2) no GPS guidance available, (3) no roaming unit system, and (4) small districts with very small streets that do not allow cars to enter them. This of course falls short of the international standard of care where the ETA in a metropolitan area should not exceed 15 min at most. The SRC response time would obviously improve by increasing the number of stations and the number of ambulance units that are covering the city of Jeddah. Most people are already aware that the EMS coverage in terms of population or in terms of area is not adequate and hence tend to expect the arrival of the paramedics within 30 min or so. The solution to this problem is multidisciplinary. It will require proper street and home addressing, telephone tracking, GPS usage, and increased unit coverage; however, more research should be done in this area to identify the best way to remedy this problem.

The need for the air ambulance or MEDEVAC seem to also sprout from the feeling of inadequate coverage of land units and the thought of getting through the traffic and finding the origin of the emergency call as early as possible. However, MEDEVAC comes with its own set of problems. For a start, it is much more expensive than land coverage. It requires proper case triage by the dispatcher to send the air unit to patients that really deserve it, with immediate life threatening or debilitating illnesses (e.g., trauma, AMI, CVA, etc.). It requires highly trained personnel (pilots, engineers, and medical staff). Landing facilities are needed, and hospital contact is essential to have an emergency team ready to receive the patient. Such air coverage should be reserved for out of city bound emergencies where land transport times are in excess of 30 min or in mass gathering situations such as in Makkah during *Hajj* season, where getting to and from the emergency scene is the major obstacle.

With concerns of allowing EMS personnel to attend to female patients unescorted by male family members, there is still a significant percentage of the population that value tradition over saving a patient’s life. This unfortunately can only be changed through the contribution of religious authorities, through sermons, and proper religious education, since nothing can break cultural tradition except religious law. Unfortunately, many people mix culture and tradition with actual religious doctrine, but they are not the same. For the Muslims, the only way to change misguided cultural belief, which is usually geographically inherited, is through proper religious education and teaching of the general public that such traditions are not of Islam. It is the Islamic law that “Necessity Knows No Law,” meaning that during an emergency, all restrictions that do apply in most circumstances are put aside until the emergency is resolved.

As far as the expectations of the public of what the SRC should do when responding to an emergency call, one in three people still think and expect that it is the job of the EMS personnel to cater to all their medical transporting need, regardless of presence or absence of an emergency. This puts enormous stress on a system that is already stretched too far, trying to cover so many people living in such a wide area. Again, education and public awareness is the key to informing the public that the EMS system is only for emergency cases, not to take bedridden patients to their OPD follow-ups or pick up patients that have already died at their homes [4]. That, and administrative policy that allows the SRC to assess the situation on the scene, with or without radio contact with the operation center, will go a long way to solving the problem of unnecessary or unwarranted medical transport.

The majority of people still think that the EMS system should be entirely funded by the government and that they should not contribute to that. Only one in three people thinks that if he/she were to contribute to the EMS funding, that will lead to better community satisfaction and higher quality of service. Instituting a small monetary amount per person to be paid annually or bi-annually might very well bring about needed changes such as new cars, stations, training, etc., and it would certainly provide funding for improvements that would be supplementary rather than integral towards improving the quality of service.

The subgroup analysis done on those that have made use of the SRC service before showed that the majority of questions asked in this survey were not influenced by whether the person had a previous encounter with the EMS system or not. This is reassuring that most of the answers obtained from both groups were valid to be combined together. It is understandable that people who have called the emergency dispatcher before should remember the number. The preference of having a unified EMS dispatcher was the only question where having a previous SRC encounter mattered. The most common scene time noted was about 15 min, and this correlated with the fact that most people expect the paramedics to treat the patient and not just scoop them up and go. Overall, it is the opinion of the EMSAT research group that the SRC is doing a good job as far as what the general public perceives, and expects, even though it may be true that the public may have poor knowledge of what to expect of the EMS system [5]. It is true that there is a large room for improvement in almost every aspect of this system, medical, public awareness, municipal, legislative, administrative, and educational, but at the end of the day, the general public seems to be understanding of the shortcomings of the EMS system in the Kingdom of Saudi Arabia’s western region. There is a great need to increase the public awareness of what the job of an EMS system should be, and what should be expected of it, so as to increase the satisfaction of the community of their service.

## Additional file

Additional file 1:
**The EMS public awareness questionnaire.**
