# Supplementary material for: Public awareness of the EMS system in Western Saudi Arabia: identifying the weakest link
Source: Int J Emerg Med. 2015 Sep 7;8:35. doi: 10.1186/s12245-015-0070-7 (PMC4561947; doi:10.1186/s12245-015-0070-7)
Supplement: Additional file 1: — The EMS public awareness questionnaire. [file 12245_2015_70_MOESM1_ESM.pdf]

Appendix A

The EMS public awareness questionnaire

Questionnair Part 1: for the General Public:

Age □30-15 □45-30 -45□75 +75 □ □ male □Female □ Saudi □ Other

1. Do you know the number of the SRC EMS dispatcher? (997) □ Yes □No
2. Have you ever called the EMS dispatcher and asked for an ambulance? □ Yes □No
3. If you were to call the SRC from you home now, how long do you think it would take them to arrive?
   □ 10mins □15mins □ 30 mins □1 hr.
4. Do you think that having a single EMS dispatcher number is better, or a number for each EMS service is better?
   □ Unified □Multiple
5. Do you think it is part of the paramedic's duty to treat patients at the scene or just to transport them?
   □ Treat □Transport
6. Do you think that the current EMS coverage for the City of Jeddah is adequate? □ Yes □No
7. Do you think we need the MEDEVAC (air ambulance) at this stage? □ Yes □No
8. Do you trust the SRC to handle your family members in a medical emergency? □ Yes □No
9. If a patient dies at their home who do you call? □ SRC □Municipal service □ Police
10. Do you think that a male paramedic should be allowed to respond to a female medical emergency, in the absence of a family male escort? □ Yes □No
11. Do you think that the SRC paramedics should have the right to refuse transport to patients who are stable and, not in a medical emergency? □ Yes □No
12. Do you think that an annual sum should be paid by the general public to fund the SRC to improve the service quality? □ Yes □No
13. If Q 12 was Yes, how much would you be willing to pay annually? □ SR50 □SR100 □ SR150 □SR200
14. What would you do if you saw an ambulance coming up behind you on the road?
    □ I would immediately get out of the way
    □ Move out of the way only if lights and sirens are on
    □ Move only if the ambulance is directly behind me
    □ I won't move an ambulance should use the emergency lane, not the middle of the road
15. What do you think of the SRC service overall?
    □Poor □Below average □ Average □Good □ Excellent

Questionnaire part 2: only for those who have had previous encounters with the SRC:

1. How long did it take for an ambulance to show up after the emergency call was placed?
   □ < 15 mins □< 30 mins □< 1 hr. □Didn't arrive & had to call again!
2. Were the paramedics in uniform? □ Yes □No
3. Did they use gloves to handle the patient? □ Yes □No
4. How long did the paramedics spend at the scene?
   □ 5 mins □10 mins □ 15mins □≥30mins
5. Did they treat the patient at the scene or just transported them? □ Treated □Just transported
6. Did you think that the SRC did a good job, or could they have done things better?
   □ Did a good job □Could've done better
